# Association between systemic inflammation response index and risk of major adverse cardiovascular events in adults with and without metabolic syndrome: a prospective cohort study in Shanghai, Pudong

**DOI:** 10.3389/fendo.2026.1846528

**Published:** 2026-06-16

**Authors:** Qiqi Meng, Juzhong Ke, Xiaonan Wang, Hua Qiu, Qingping Liu, Jiaojiao Gao, Jiahui Song, Yang Liu, Qian Xu, Mengyao Wu, Bo Huang, Yuling Qian, Lin Song, Xiaonan Ruan, Kang Wu, Yi Zhou

**Affiliations:** Shanghai Pudong New Area Center for Disease Control and Prevention (Shanghai Pudong New Area Health Supervision Institute), Pudong Institute of Preventive Medicine of Fudan University, Shanghai, China

**Keywords:** cardiovascular risk, inflammation, MACE, prospective cohort study, SIRI

## Abstract

**Objective:**

To investigate the prognostic value of the systemic inflammation response index (SIRI) for major adverse cardiovascular events (MACE) among adults with metabolic syndrome (MS), and to determine whether MS status modifies the association between SIRI and incident MACE.

**Methods:**

This prospective cohort study enrolled 3,198 participants from Pudong New Area, Shanghai, with a median follow-up of 43 months. Baseline characteristics were compared across SIRI quartiles stratified by MS status. Multivariable Cox proportional hazards regression models were constructed to evaluate the independent association between SIRI and MACE. Restricted cubic spline (RCS) analysis was performed to examine the dose–response relationship. Interaction analyses were conducted to assess effect modification by key metabolic components.

**Results:**

Of 3,198 participants, 1,318 had MS and 1,880 did not. Higher SIRI quartiles were associated with unfavorable metabolic profiles in both groups. Compared with non-MS participants in the lowest SIRI quartile, participants with MS in the highest SIRI quartile exhibited the greatest risk of MACE (HR = 2.33, 95% CI: 1.64–3.30, P < 0.001). Among participants with MS, the highest quartile demonstrated a 38% elevated risk compared with the lowest quartile (HR = 1.38, 95% CI: 1.08-1.77, P = 0.01). RCS analysis indicated a linear positive association between SIRI and MACE risk among participants with MS (P for overall association = 0.02). A significant non-linear interaction was identified between SIRI and fasting plasma glucose (P for interaction = 0.04).

**Conclusion:**

Elevated SIRI is independently associated with an increased risk of incident MACE, and MS status significantly modifies this association. SIRI represents a promising and readily available biomarker for cardiovascular risk stratification, especially among individuals with MS. Combined interventions targeting systemic inflammation and glycemic control may reduce the risk of MACE in this high-risk population.

## 1 Introduction

Cardiovascular disease (CVD) continues to be the leading cause of mortality and morbidity worldwide, imposing a substantial socioeconomic and clinical burden on public health systems (1). Metabolic syndrome (MS), a cluster of interconnected metabolic disorders including central obesity, hyperglycemia, hypertension, and dyslipidemia, is widely recognized as a major modifiable risk factor for atherosclerotic cardiovascular disease (2, 3). The pathophysiological mechanisms linking MS to adverse cardiovascular outcomes are complex and multifactorial, with chronic low-grade inflammation regarded as a central mechanistic driver (4). Sustained systemic inflammation promotes endothelial dysfunction, accelerates atherogenesis, and induces a prothrombotic state, all of which contribute directly to the development and progression of major adverse cardiovascular events (MACE) (5, 6).

In recent years, considerable research attention has focused on identifying simple, inexpensive, and routinely available inflammatory biomarkers to improve cardiovascular risk prediction across diverse populations. The systemic inflammation response index (SIRI), calculated as (neutrophil count × monocyte count)/lymphocyte count, is a novel composite inflammatory marker that integrates the balance between pro-inflammatory and anti-inflammatory immune cell subsets (7, 8). Unlike single inflammatory markers such as C-reactive protein or interleukin-6, SIRI captures the coordinated dynamics of distinct immune cell populations that jointly regulate inflammatory cascades during atherogenesis (9). Neutrophils and monocytes promote endothelial injury and plaque progression, whereas lymphocytes exert anti-inflammatory and vascular-protective effects (10, 11).

Emerging epidemiological evidence has linked elevated SIRI to adverse cardiovascular outcomes in various clinical populations, including patients with ST-elevation myocardial infarction, hypertension, type 2 diabetes, and ischemic stroke (12–14). However, most existing studies have focused on specific disease cohorts or have not stratified analyses by MS status. Consequently, the predictive value of SIRI for incident MACE among individuals with MS, who are characterized by chronic inflammation and inherently elevated cardiovascular risk remains poorly defined. Furthermore, whether key metabolic components (e.g., fasting plasma glucose, blood pressure, lipid profile) interact with SIRI to modify cardiovascular risk in patients with MS has not been comprehensively explored.

To address these critical evidence gaps, we conducted a prospective population-based cohort study in Pudong New Area, Shanghai, to evaluate the association between SIRI and incident MACE, with a specific focus on individuals with MS. We hypothesized that: (1) elevated baseline SIRI is independently associated with an increased risk of MACE among participants with MS; and (2) SIRI interacts with metabolic abnormalities, particularly hyperglycemia, to amplify cardiovascular risk. The findings of this study may provide novel insights into inflammation−guided cardiovascular risk stratification and inform the development of targeted preventive strategies for adults with MS.

## 2 Methods

### 2.1 Study population

**Figure 1:**
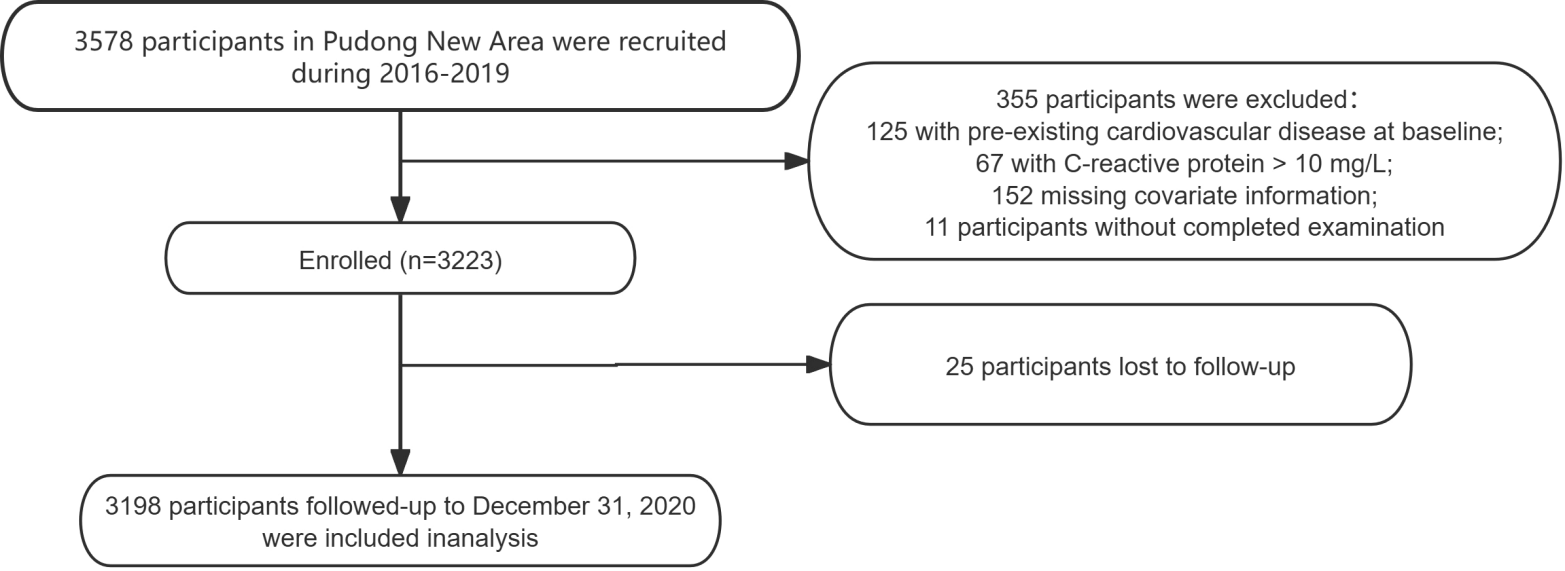
Flow diagram of participant recruitment, exclusion, follow-up, and analysis in this prospective cohort study.

In this prospective cohort study, participants were recruited from the Pudong New Area cohort in Shanghai, China, between 2016 and 2019. The detailed participant recruitment and screening procedure is presented in **Figure 1**. Inclusion criteria were as follows: (1) age ≥18 years; (2) availability of complete baseline data on MS components, SIRI-related immune cell counts, and demographic characteristics; (3) willingness to participate in follow-up assessments. Exclusion criteria included: (1) presence of acute inflammation or infection at baseline, defined as fever or C-reactive protein >10 mg/L; (2) pre-existing cardiovascular disease at baseline; (3) incomplete follow-up data. A total of 3,198 participants were included in the final analysis, with 1,318 participants classified as having MS and 1,880 as non-MS.

### 2.2 Follow-up and outcome ascertainment

Participants were followed for a median of 43 months (interquartile range [IQR]: 42–45 months). The primary endpoint was incident MACE, defined as a composite of non-fatal myocardial infarction, non-fatal stroke, and cardiovascular death. The follow-up was completed via telephone interviews, medical record review, and community-based health surveillance. In addition, we performed individual record cross-matching across multiple systems, including the electronic health records (EHR) system and the regional hospital diagnosis and treatment system, to further clarify the participants’ outcome information. The follow-up period extended from the baseline examination to the date of the first MACE, death, or December 31, 2020, whichever occurred first.

### 2.3 Definitions and measurements

Metabolic syndrome was defined according to the Guidelines for the Prevention and Treatment of Type 2 Diabetes Mellitus in China (2024 Edition). Participants were classified as having MS if they met ≥ 3 of the following 5 criteria: Central obesity: waist circumference ≥ 90 cm (men) or ≥ 85 cm (women); Hyperglycemia: fasting plasma glucose ≥ 6.1 mmol/L, 2-hour postload glucose ≥ 7.8 mmol/L, or previously diagnosed diabetes; Hypertension: systolic blood pressure ≥ 130 mmHg, diastolic blood pressure ≥ 85 mmHg, or previously diagnosed hypertension; Hypertriglyceridemia: fasting triglycerides ≥ 1.70 mmol/L; Low HDL-cholesterol: fasting HDL-C < 1.04 mmol/L.

SIRI calculation: Peripheral venous blood samples were collected at baseline and analyzed using a Beckman Coulter LH 750 Hematology Analyzer. SIRI was computed as: SIRI = (Neutrophil count × Monocyte count)/Lymphocyte count.

Participants were stratified by MS status (MS vs non-MS), and further divided into quartiles of SIRI within each stratum, yielding 8 mutually exclusive subgroups for analyses.

### 2.4 Baseline data

Demographic data (age, sex, education level, marital status), metabolic parameters (waist circumference, systolic blood pressure [SBP], diastolic blood pressure [DBP], fasting plasma glucose [FPG], triglycerides [TG]), and inflammatory indices were collected at baseline. Health-related behaviors (physical activity, prolonged sitting time, smoking status, alcohol use), medication history, and prevalence of hypertension were also recorded.

### 2.5 Covariables

Covariates were collected through standardized questionnaires and clinical assessments. Demographic information included age, sex, marital status and education. Education level was categorized into 2 groups: completed ≥ 9 years of education and not completed, marital status into 2 groups (married and other). Health behaviors included physical activity (physical activity defined as ≥ 1 sports session/week maintained for ≥ 5 years vs inactive) and prolonged sitting time (daily accumulated sedentary time ≥ 8 hours vs < 8 hours). Cardiovascular risk factors included smoking status (current smoker defined as ≥1 cigarette/day for ≥6 months vs non-smoker), alcohol consumption (regular drinker defined as ≥3 drinking occasions/week for ≥6 months vs non-drinker) and medication use (use of any of the three medications including antihypertensive medications, lipid-lowering medications, anti-angina or anti-arrhythmic cardiovascular medications) compared with no medication use. Participants with missing data on covariates were excluded from analysis.

### 2.6 Statistical analysis

Statistical analyses were performed using R software (version 4.2.5). Continuous variables were presented as median (IQR) and compared using the Kruskal-Wallis test. Categorical variables were presented as n (%) and compared using the chi-square test.

Multivariable Cox proportional hazards models were used to estimate hazard ratios (HRs) and 95% confidence intervals (CIs) for the association between SIRI and incident MACE. Models were adjusted for age, sex, education, marital status, physical activity, prolonged sitting, smoking, alcohol use and medication. Analyses were performed in the total population and stratified by MS status. Proportional hazards assumption was tested using Schoenfeld residuals. Age violated the assumption in both the MS and non-MS populations. Accordingly, an age−time interaction term was introduced into the Cox models for these two subgroups to correct the violation. The corresponding Schoenfeld residual plots for the total, MS, and non-MS populations are provided in the **Supplementary Materials**.

Restricted cubic spline (RCS) analysis with three knots (10th, 50th, 90th percentiles) was used to evaluate the dose-response relationship between continuous SIRI and MACE risk among participants with MS. This knot selection strategy was adopted based on conventional statistical recommendations and previous epidemiological studies to ensure adequate curve fitting and avoid overfitting. We then performed tests for linear trend and non-linearity.

Interaction analyses were conducted to examine effect modification by fasting plasma glucose, SBP, TG, and waist circumference. The proportional hazards assumption was verified using Schoenfeld residuals. A two-sided P < 0.05 was considered statistically significant.

## 3 Results

### 3.1 Baseline characteristics stratified by MS and SIRI

A total of 3,198 participants were stratified into 8 subgroups based on MS status and SIRI quartiles. Among the 1,318 participants with MS, each SIRI quartile included approximately 330 individuals. Among the 1,880 non-MS participants, each quartile included approximately 470 individuals.

As presented in **Table 1**, significant differences in baseline characteristics were observed among subgroups (all P < 0.05 except for educational level, physical activity, marital status and prolonged sitting). Participants with MS were older (61–63 years) than those without MS (59–60 years). Within both MS and non-MS groups, higher SIRI quartiles were associated with a higher proportion of men, less favorable metabolic profiles, greater waist, fasting glucose, blood pressure, higher triglyceride levels, smoking and alcohol consumption rates, and a greater prevalence of hypertension and medication use. SIRI values exhibited clear gradient separation across quartiles: Q1: 0.28-0.32; Q2: 0.43-0.50; Q3: 0.60-0.68; Q4: 0.94-1.03.

**Table 1:** Baseline characteristics of study participants stratified by metabolic syndrome status and SIRI quartiles.

Variable	Overall	MS				Non-MS				P-value
		MS, SIRI Q1	MS, SIRI Q2	MS, SIRI Q3	MS, SIRI Q4	Non-MS, SIRI Q1	Non-MS, SIRI Q2	Non-MS, SIRI Q3	Non-MS, SIRI Q4	
N	3198	330	330	329	329	470	470	470	470	
Age, years	60.5 (10.4)	61.3 (8.5)	61.5 (10.0)	62.3 (10.1)	62.6 (9.9)	59.8 (8.6)	59.6 (10.8)	59.4 (11.3)	59.2 (12.3)	<0.001
Waist, cm	83.3 (9.2)	88.4 (8.1)	87.9 (7.9)	89.9 (8.0)	90.0 (8.1)	78.1 (7.9)	78.6 (7.3)	80.1 (7.4)	80.5 (7.5)	<0.001
FPG, mmol/L	6.3 (1.8)	6.9 (2.2)	6.8 (1.9)	6.9 (2.0)	7.1 (2.6)	5.7 (1.1)	5.9 (1.5)	5.8 (1.4)	5.8 (1.3)	<0.001
SBP, mmHg	140.4 (20.3)	147.5 (18.3)	148.0 (18.9)	148.7 (19.3)	147.6 (19.2)	133.8 (18.4)	135.7 (19.6)	135.7 (20.4)	134.9 (19.4)	<0.001
DBP, mmHg	84.6 (10.6)	88.8 (9.6)	88.5 (9.7)	89.7 (10.3)	88.2 (10.5)	81.4 (9.9)	82.0 (9.6)	81.9 (10.4)	81.5 (10.2)	<0.001
TG, mmol/L	1.5 (1.3)	2.3 (2.1)	2.2 (1.3)	2.0 (1.6)	2.3 (1.7)	1.0 (0.5)	1.1 (0.5)	1.1 (0.4)	1.1 (0.5)	<0.001
SIRI	0.6 (0.3)	0.3 (0.1)	0.5 (0.0)	0.7 (0.1)	1.0 (0.2)	0.3 (0.1)	0.4 (0.0)	0.6 (0.1)	0.9 (0.2)	<0.001
Male, n (%)	1129 (35.3)	80 (24.2)	128 (38.8)	152 (46.2)	200 (60.8)	73 (15.5)	118 (25.1)	169 (36.0)	209 (44.5)	<0.001
≥9 years of education, n (%)	2348 (73.4)	249 (75.5)	251 (76.1)	255 (77.5)	251 (76.3)	346 (73.6)	336 (71.5)	345 (73.4)	315 (67.0)	0.018
Physical activity, n (%)	2485 (77.7)	256 (77.6)	265 (80.3)	247 (75.1)	243 (73.9)	384 (81.7)	362 (77.0)	358 (76.2)	370 (78.7)	0.148
Married, n (%)	2912 (91.1)	299 (90.6)	312 (94.5)	302 (91.8)	292 (88.8)	425 (90.4)	431 (91.7)	434 (92.3)	417 (88.7)	0.103
Prolonged sitting, n (%)	577 (18.0)	52 (15.8)	59 (17.9)	65 (19.8)	73 (22.2)	80 (17.0)	77 (16.4)	73 (15.5)	98 (20.9)	0.123
Hypertension, n (%)	2432 (76.0)	315 (95.5)	313 (94.8)	312 (94.8)	314 (95.4)	283 (60.2)	293 (62.3)	304 (64.7)	298 (63.4)	<0.001
Smoking, n (%)	582 (18.2)	35 (10.6)	53 (16.1)	75 (22.8)	132 (40.1)	31 (6.6)	60 (12.8)	81 (17.2)	115 (24.5)	<0.001
Alcohol use, n (%)	415 (13.0)	30 (9.1)	49 (14.8)	49 (14.9)	78 (23.7)	26 (5.5)	41 (8.7)	65 (13.8)	77 (16.4)	<0.001
Medication, n (%)	1529 (47.8)	221 (67.0)	206 (62.4)	219 (66.6)	203 (61.7)	173 (36.8)	169 (36.0)	177 (37.7)	161 (34.3)	<0.001

### 3.2 Cox regression analysis based on metabolic syndrome status

The results of multivariable Cox proportional hazards regression models evaluating the association between SIRI quartiles and the risk of MACE in the total cohort and stratified by MS status are presented in **Table 2**. Two regression models were constructed: Model 1 was the unadjusted crude model, while Model 2 was fully adjusted for potential confounders including age, gender, educational attainment, marital status, prolonged sitting time, physical activity, smoking, alcohol consumption, and medication history.

**Table 2:** Cox regression results for MACE risk by SIRI quartiles and MS status.

Characteristic	Model 1 (Crude)		Model 2 (Adjusted)	
	HR (95% CI)	P value	HR (95% CI)	P value
Total Population (N = 3198, 523 events)				
Non-MS, SIRI Q1	1.00 (Reference)	–	1.00 (Reference)	–
Non-MS, SIRI Q2	1.79 (1.27–2.54)	0.001	1.71 (1.21–2.43)	0.003
Non-MS, SIRI Q3	1.49 (1.03–2.14)	0.034	1.35 (0.93–1.95)	0.115
Non-MS, SIRI Q4	1.62 (1.14–2.31)	0.007	1.39 (0.97–1.99)	0.073
MS, SIRI Q1	1.71 (1.18–2.48)	0.005	1.73 (1.19–2.52)	0.004
MS, SIRI Q2	1.67 (1.16–2.41)	0.006	1.57 (1.08–2.28)	0.018
MS, SIRI Q3	2.20 (1.55–3.13)	<0.001	1.89 (1.32–2.71)	<0.001
MS, SIRI Q4	2.90 (2.07–4.07)	<0.001	2.33 (1.64–3.30)	<0.001
Non-MS Population (n = 1880, 256 events)				
SIRI Q1	1.00 (Reference)	–	1.00 (Reference)	–
SIRI Q2	1.83 (1.30–2.60)	<0.001	1.83 (1.28–2.60)	<0.001
SIRI Q3	1.56 (1.08–2.25)	0.018	1.41 (0.97–2.06)	0.073
SIRI Q4	1.65 (1.16–2.36)	0.005	1.46 (1.01–2.12)	0.043
P for trend		<0.001		0.208
MS Population (n = 1318, 267 events)				
SIRI Q1	1.00 (Reference)	–	1.00 (Reference)	–
SIRI Q2	1.36 (1.07–1.74)	0.014	1.31 (1.02–1.67)	0.036
SIRI Q3	1.42 (1.11–1.82)	0.005	1.23 (0.96–1.57)	0.11
SIRI Q4	1.68 (1.32–2.12)	<0.001	1.38 (1.08–1.77)	0.01
P for trend		<0.001		0.026

Model 1 is a crude model without any adjustment. Model 2 is adjusted for age, gender, marital status, education level, prolonged sitting time, physical activity, smoking status, alcohol drinking, and medication history. The proportional hazards assumption was violated for age in both non-MS and MS populations; therefore, time-dependent interaction terms of age were further incorporated into the Cox models to correct the violation.

HR, hazard ratio; CI, confidence interval; SIRI, systemic inflammation response index; MS, metabolic syndrome; MACE, major adverse cardiovascular event.

In the total population (N = 3,198), compared with metabolically healthy participants in the lowest SIRI quartile (non-MS, SIRI Q1), participants with MS in the highest SIRI quartile (MS, SIRI Q4) exhibited the most significantly elevated risk of MACE after full adjustment (HR = 2.33, 95% CI: 1.64-3.30, P<0.001). Even participants with MS in the lowest SIRI quartile showed a significantly increased MACE risk relative to the reference group (HR = 1.73, 95% CI: 1.19-2.52, P = 0.004).

Stratified analyses revealed divergent patterns by MS status. Among participants with MS (n = 1, 318), higher SIRI levels were monotonically associated with an increased risk of MACE after full adjustment (P for trend=0.026), with the highest quartile demonstrating a 38% elevated risk compared with the lowest quartile (HR = 1.38, 95% CI: 1.08-1.77, P = 0.01). In contrast, among participants without MS (n = 1,880), the magnitude of the association between SIRI and MACE risk was strongest in the SIRI Q2 group, which exhibited a significant increase in MACE risk (HR = 1.83, 95% CI: 1.2-2.60, P < 0.001); the associations were gradually attenuated in the higher quartiles, with marginally significant risks observed in SIRI Q3 (HR = 1.41, 95% CI: 0.97–2.06, P = 0.073) and SIRI Q4 (HR = 1.46, 95% CI: 1.01–2.12, P = 0.043). Consistently, the linear trend across SIRI quartiles was no longer significant in the non-MS population after multivariate adjustment (P for trend = 0.208).

### 3.3 Assessment of proportional hazards assumption

The proportional hazards assumption for all Cox regression models was formally tested using Schoenfeld residuals. After adjusting for all covariates, residual plots were generated for the total population, MS subgroup, and non-MS subgroup, and the corresponding plots were presented in the supplementary materials. In the total population model, the global chi-square test indicated no significant violation (χ² = 19.438, df=16, P = 0.25). Among non-MS participants, the global test was non-significant (χ² = 14.58, df=12, P = 0.27), but age violated the assumption (P = 0.03). Notably, among participants with MS, the proportional hazards assumption was fully satisfied, with a non-significant global test (χ²=13.56, df=12, P = 0.33) but age also violated the assumption (P = 0.023); therefore, an age-time interaction term was incorporated into the analyses to address this violation. Importantly, the SIRI variable met the proportional hazards assumption consistently across all analytic models (total population: P = 0.72; non-MS: P = 0.99; MS: P = 0.59), supporting the robustness of the estimated HRs as average effect measures over the follow-up period. Detailed test results are provided in the **Supplementary Materials**.

### 3.4 Restricted cubic spline analysis

**Figure 2:**
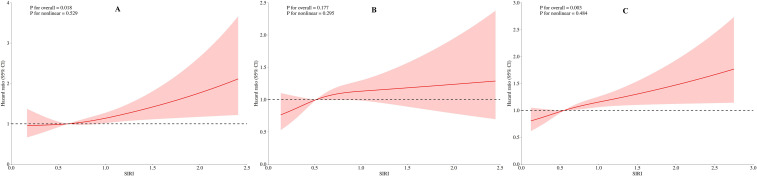
Dose–response relationship between systemic inflammation response index (SIRI) and major adverse cardiovascular events (MACE) risk. **(A)** Participants with metabolic syndrome (MS); **(B)** Participants without MS; **(C)** Total study population. All analyses were adjusted for age, gender, marital status, education level, prolonged sitting time, physical activity, smoking status, alcohol drinking, and medication history.

Restricted cubic spline (RCS) regression was performed to characterize the dose–response relationship between continuous SIRI and MACE risk, stratified by MS status (**Figure 2**). After full covariate adjustment, distinct association patterns emerged across subgroups.

Among participants with MS (Panel A), RCS analysis confirmed a significant linear positive association between SIRI and MACE risk (P for overall association = 0.02). The hazard curve crossed the reference line (HR = 1.0) at a SIRI value of approximately 0.6 and rose progressively with increasing SIRI; however, the non-linear component was not statistically significant (P for non-linearity = 0.53). Among participants without MS (**Figure 2B**), the overall association was non-significant (P = 0.18), with HR values remaining close to unity across most of the SIRI distribution and only a slight elevation at high SIRI levels (P for non-linearity = 0.30). In the total population (**Figure 2C**), a significant monotonic linear association was observed (P = 0.003), with no evidence of non-linearity (P = 0.45). The HR was below 1.0 at SIRI values below 0.6, crossed unity at approximately 0.6, and increased continuously thereafter. Collectively, these results indicate that MS status modifies the association between SIRI and incident MACE, with a robust positive linear relationship in participants with MS.

### 3.5 Interaction between SIRI and metabolic components in MS patients

Considering that the association of SIRI with MACE risk was robust and linearly positive among individuals with MS, we further explored potential effect modification of this relationship by key metabolic components in the MS population. A significant non-linear interaction was identified between SIRI and fasting plasma glucose (FPG) in predicting MACE (P for interaction = 0.04; P for non-linear interaction = 0.03), which was driven primarily by glucose levels (P = 0.02). No statistically significant interactions were observed between SIRI and systolic blood pressure (P = 0.53), TG (P = 0.24), or waist circumference (P = 0.13).

**Figure 3:**
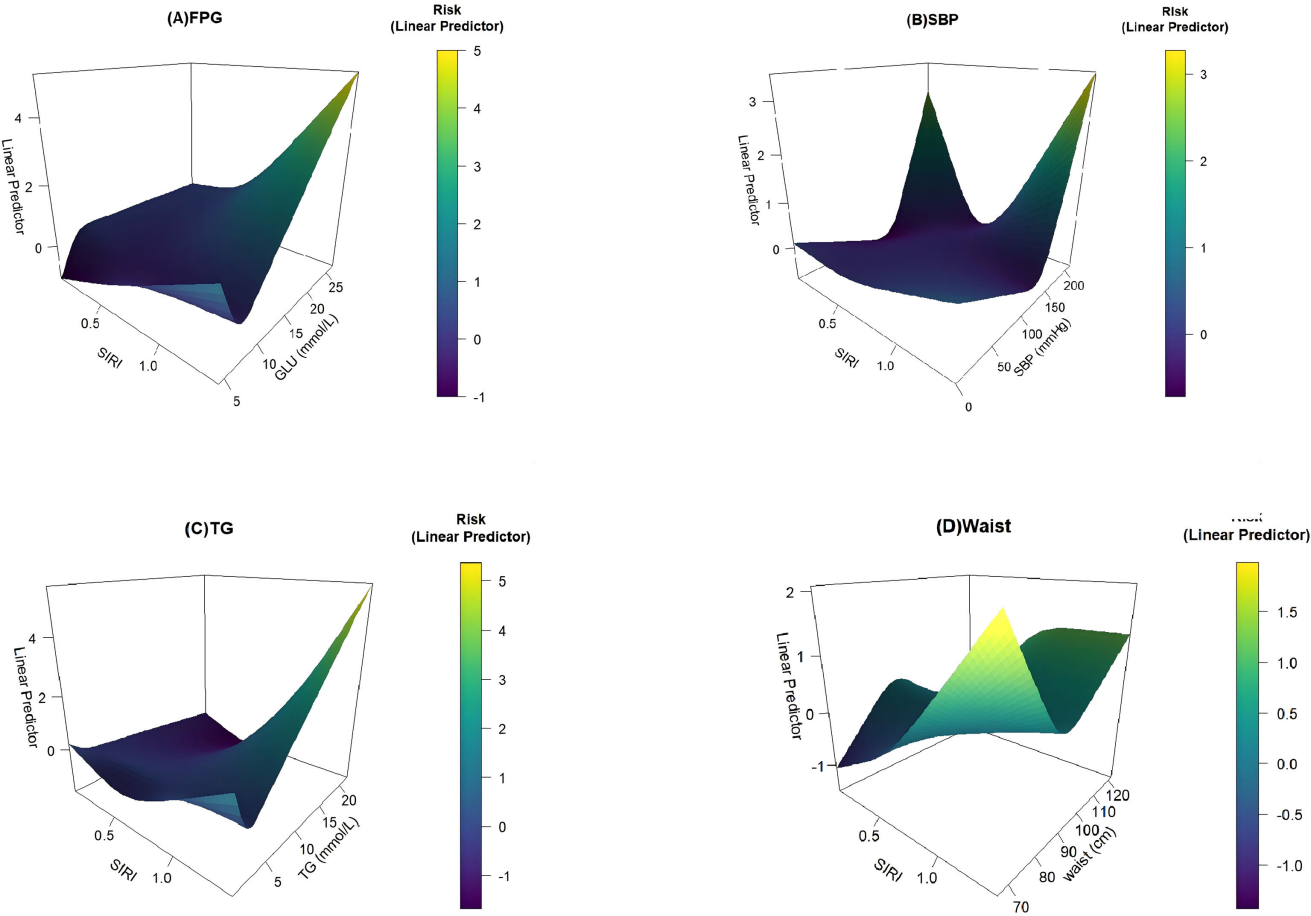
Three-dimensional surface plots illustrating the joint effect of SIRI and key metabolic components on MACE risk in patients with MS. Subplot titles are as follows: **(A)** Joint effect of SIRI and fasting plasma glucose (FPG); **(B)** Joint effect of SIRI and systolic blood pressure (SBP); **(C)** Joint effect of SIRI and triglycerides (TG); **(D)** Joint effect of SIRI and waist circumference. All analyses were adjusted for age, gender, marital status, education level, prolonged sitting time, physical activity, smoking status, alcohol drinking, and medication history.

Marginal effect plots confirmed that the adverse impact of elevated SIRI on MACE risk was markedly amplified among participants with MS and higher glucose concentrations (**Figure 3**). These findings indicate that the cardiovascular risk attributable to systemic inflammation in MS is not uniform, but is strongly potentiated by concomitant hyperglycemia. Participants with MS exhibiting combined elevations in SIRI and FPG thus represent a high-risk subgroup that may benefit from intensified preventive strategies.

## 4 Discussion

In this community-based cohort study, we identified a significant linear association between elevated SIRI levels and the incidence of MACE events among participants with MS. We also detected a significant interaction between SIRI and FPG for MACE risk in the MS population. These findings demonstrate that SIRI has good predictive value for the incidence of MACE in the MS population, implying that it could serve as a tool for risk stratification of community residents. The results of this study offer insights into specific populations for optimizing health promotion strategies in Chinese communities.

Emerging evidence regarding the association between systemic inflammatory indices and cardiovascular outcomes has largely originated from Western cohorts such as the National Health and Nutrition Examination Survey (NHANES) (15–17). However, these findings cannot be directly generalized to Chinese populations due to marked differences in lifestyle, dietary patterns, genetic background, and the prevalence of metabolic and cardiovascular diseases. The present study was based on a community-dwelling middle-aged and elderly cohort from Pudong New Area, Shanghai, which well represents the characteristics of urban Chinese residents. In this context, our results provide ethnicity-specific epidemiological evidence and complement existing data from Western populations.

Our findings align with prior evidence linking elevated SIRI to MACE. A study of the population with cardiovascular-renal-metabolic (CKM) syndrome, utilizing data from the UK Biobank, consistently demonstrated a significant association between elevated SIRI and the risk of developing cardiovascular disease (CVD) (18). Furthermore, two additional studies revealed a substantial correlation between SIRI and MACE following myocardial infarction, as well as the comprehensive clinical risk factors in patients with acute myocardial infarction (AMI). SIRI may serve as a more accurate and cost-effective indicator for predicting the long-term prognosis of patients with ST-segment elevation myocardial infarction (STEMI) (19, 20).

Notably, the dose-response relationship between SIRI and MACE events exhibits distinct patterns across different populations. In this study, we detected a significant linear correlation between the elevation of SIRI levels and the heightened risk of MACE events. This outcome aligns with the findings of a clinical study involving patients with primary biliary cholangitis (PBC), and two studies based on NHANES have also validated this result (6, 21, 22). In patients with MS, a higher SIRI value is associated with a greater probability of MACE. Our findings indicate that when the SIRI value surpasses 0.6, the risk of MACE increases significantly (**Figure 2A**).

This prospective cohort study investigated the association between SIRI and MACE risk in 3198 adults from Shanghai Pudong, stratified by MS status, and explored the interaction between SIRI and key metabolic components in MS patients. Our findings revealed several important observations: MS patients had significantly higher SIRI levels and more unfavorable metabolic and inflammatory profiles compared to non-MS individuals; elevated SIRI was associated with increased MACE risk in total population, with the highest risk observed in MS patients with the highest SIRI quartile; the association between SIRI and MACE risk differed by MS status, showing a steady linear increasing trend in the MS subgroup while a non-monotonic and attenuated trend in the non-MS subgroup; a linear dose-response relationship between SIRI and MACE risk was observed in MS patients; and SIRI interacted with FPG to increase MACE risk in MS patients, while no significant interactions were found with other metabolic components.

### 4.1 Key findings and interpretation

Stratified analysis demonstrated a differential association between the SIRI and MACE according to MS status. Among individuals with MS, elevated SIRI levels were consistently associated with higher MACE risk: compared with the lowest SIRI quartile, those in the highest quartile had a 38% increased risk (HR = 1.38, 95% CI:1.08-1.77; P = 0.01). This observation is consistent with prior studies reporting SIRI as an independent prognostic biomarker for cardiovascular outcomes across heterogeneous populations (6, 18, 20–25).

RCS analysis further demonstrated a statistically significant association between SIRI and MACE risk in patients with MS. The risk of MACE increased monotonically with SIRI above approximately 0.6. This pattern suggests a potential threshold effect of SIRI in patients with MS. The approximate value of this threshold is 0.6, which is an exploratory observational finding from the present study rather than a clinically validated one. Moreover, higher SIRI values were associated with increased MACE risk. In the overall population, the dose-response relationship between SIRI and MACE was linear; however, no statistically significant association was observed in individuals without MS. These findings collectively support a modifying role of MS status on the SIRI-MACE association.

Importantly, we observed a statistically significant nonlinear interaction between the SIRI and FPG in predicting MACE among patients with MS. Specifically, the adverse association of elevated SIRI with MACE risk was markedly amplified at higher FPG levels, suggesting that the co-occurrence of systemic inflammation and hyperglycemia defines a high-risk phenotype. This interaction may be attributable to synergistic effects of inflammation and hyperglycemia on endothelial dysfunction and atherosclerosis progression (26). Hyperglycemia induces oxidative stress and the production of pro-inflammatory cytokines, thereby further amplifying the pro-inflammatory activity of neutrophils and monocytes as reflected by an elevated SIRI (27, 28). In contrast, no statistically significant interaction was observed between SIRI and other metabolic components including SBP, TG, and waist circumference, suggesting that hyperglycemia may serve as a key metabolic mediator linking systemic inflammation and cardiovascular risk in individuals with metabolic syndrome.

### 4.2 Comparison with previous studies

Our findings are partially consistent with prior studies investigating the SIRI and cardiovascular outcomes. For instance, a single-center cohort study involving 1,312 patients with coronary artery disease reported that a higher SIRI was associated with an increased incidence of 30-day MACE among patients with ST-segment elevation myocardial infarction (STEMI). These results suggest that SIRI may serve as a potential prognostic indicator for short-term outcomes in STEMI patients (12). Another study conducted among individuals with hypertension indicated an association between higher SIRI and increased CVD-related mortality (13). However, these prior studies did not stratify analyses by MS status; thus, the differential associations observed in our study provide novel insights. A recent study by Huang et al. based on the NHANES database, reported an association between the SIRI and cardiovascular mortality risk among individuals with cardiovascular-kidney-metabolic syndrome (29). However, the aforementioned study employed a cross-sectional design and did not assess potential interactions between the SIRI and metabolic parameters. In contrast, our prospective cohort study complemented by comprehensive stratified and interaction analyses addresses this methodological limitation. Specifically, we provide robust evidence on the prognostic utility of SIRI in patients with MS, including its differential associations across glycemic status and formal evaluation of effect modification by hyperglycemia.

### 4.3 Clinical implication

This study yields several clinically relevant implications. First, the SIRI may serve as a simple and readily applicable prognostic marker for MACE in participants with MS. As SIRI is derived from routine peripheral blood cell counts, widely available in clinical practice, it can be feasibly incorporated into risk stratification workflows. Second, the observed dose-response relationship between SIRI and MACE risk among individuals with MS suggests that maintaining SIRI below approximately 0.6 may be associated with reduced MACE incidence, indicating that SIRI may serve as a potential modifiable target for intervention. Of note, this value merely represents an exploratory observation from the present study, rather than a clinically established threshold. Third, a statistically significant interaction between SIRI and fasting plasma glucose underscores the importance of concurrently addressing systemic inflammation and hyperglycemia in the cardiovascular risk management of patients with MS. Clinicians may consider prioritizing glycemic control in MS patients exhibiting elevated SIRI, as this combination identifies a subgroup at particularly high risk for MACE.

Moreover, our study underscores the importance of individualized cardiovascular risk assessment in patients with MS. Although MS itself is an established cardiovascular risk factor, elevated SIRI and fasting glucose levels may help stratify patients according to risk magnitude, thereby informing more targeted preventive interventions such as lifestyle modification, anti-inflammatory agents, or glucose-lowering therapies. This approach is particularly relevant in China, where the prevalence of MS continues to rise (30), and cardiovascular diseases are a leading cause of death.

### 4.4 Strengths and limitations

This study has several strengths. First, it is a prospective cohort study with a median follow-up of 43 months, which allows for the assessment of temporal relationships between SIRI and MACE risk. Second, the study population is large (3198 participants) and well-characterized, with detailed baseline data on metabolic components, immune cell counts, and potential confounders. Third, we stratified analyses by MS status and explored dose-response relationships and interactions with metabolic components, providing comprehensive insights into the role of SIRI in different subgroups. Fourth, we verified the proportional hazards assumption for Cox models, ensuring the validity of the reported HRs.

Despite these strengths, several limitations should be noted. First, the study was conducted in a single region (Shanghai Pudong), which may limit the generalizability of the findings to other populations, particularly those with different ethnic or socioeconomic backgrounds. Second, we only measured SIRI at baseline, and changes in SIRI over time may affect MACE risk. Future studies with repeated SIRI measurements obtained through data linkage with EHR and hospital laboratory information systems (LIS) are warranted to further improve the accuracy of our findings. Third, the definition of MACE did not include some cardiovascular outcomes (e.g., heart failure), which may have underestimated the total cardiovascular burden. Finally, the RCS analysis for non-linearity did not reach statistical significance in some subgroups, which may be due to limited statistical power.

## 5 Conclusion

In conclusion, elevated SIRI is independently associated with increased MACE risk in individuals with MS, with a linear dose-response relationship. The interaction between SIRI and fasting plasma glucose highlights the importance of managing both inflammation and hyperglycemia. SIRI may serve as a practical biomarker for cardiovascular risk stratification, particularly in MS patients.

## Data Availability

The original contributions presented in the study are included in the article/**Supplementary Material**. Further inquiries can be directed to the corresponding author.

## References

[1] Organization WH . Noncommunicable diseases progress monitor 2022. Geneva: World Health Organization (2022).

[2] Chinese Diabetes Society . Guideline for the prevention and treatment of diabetes mellitus in China (2024 edition). Chin J Diabetes Mellit. (2025) 17:16–139.

[3] Alberti KGMM Eckel RH Grundy SM Zimmet PZ Cleeman JI Donato KA . Harmonizing the metabolic syndrome. Circulation. (2009) 120:1640–5. doi: 10.1161/circulationaha.109.192644 19805654

[4] Saltiel AR Olefsky JM . Inflammatory mechanisms linking obesity and metabolic disease. J Clin Invest. (2017) 127:1–4. doi: 10.1172/jci92035 28045402 PMC5199709

[5] Silveira Rossi JL Barbalho SM Reverete de Araujo R Bechara MD Sloan KP Sloan LA . Metabolic syndrome and cardiovascular diseases: Going beyond traditional risk factors. Diabetes Metab Res Rev. (2021) 38(3):e3502. doi: 10.1002/dmrr.3502 34614543

[6] Cao Y Wang W Xie S Xu Y Lin Z . Joint association of the inflammatory marker and cardiovascular-kidney-metabolic syndrome stages with all-cause and cardiovascular disease mortality: a national prospective study. BMC Public Health. (2025) 25(1):10. doi: 10.1186/s12889-024-21131-2 39748335 PMC11697861

[7] Yang Z Li S Wang T Zhao X Wang F Zhang X . The role of systemic inflammatory response index in predicting myocardial infarction in patients with unstable angina. Front Cardiovasc Med. (2026) 12:1652379. doi: 10.3389/fcvm.2025.1652379 41847098 PMC12989586

[8] Marchi F Pylypiv N Parlanti A Storti S Gaggini M Paradossi U . Systemic immune-inflammation index and systemic inflammatory response index as predictors of mortality in ST-elevation myocardial infarction. J Clin Med. (2024) 13(5):1256. doi: 10.3390/jcm13051256 38592104 PMC10931789

[9] Xia Y Xia C Wu L Li Z Li H Zhang J . Systemic immune inflammation index (SII), system inflammation response index (SIRI) and risk of all-cause mortality and cardiovascular mortality: a 20-year follow-up cohort study of 42,875 US adults. J Clin Med. (2023) 12(3):1128. doi: 10.3390/jcm12031128 36769776 PMC9918056

[10] Libby P Pasterkamp G Crea F Jang I-K . Reassessing the mechanisms of acute coronary syndromes. Circ Res. (2019) 124:150–60. doi: 10.1161/circresaha.118.311098 30605419 PMC6447371

[11] Kuo IC Brassard J Zandstra PW McNagny KM . Innate lymphoid cells in the spotlight: from biomarkers to blueprint for innovative immunotherapy. Front Immunol. (2025) 16:1655730. doi: 10.3389/fimmu.2025.1655730 40963622 PMC12436429

[12] Gao H Li X Qu C . The impact of systemic inflammation response index on the prognosis of patients with ST-segment elevation myocardial infarction undergoing percutaneous coronary intervention. Rev Cardiovasc Med. (2023) 24(5):153. doi: 10.31083/j.rcm2405153 39076749 PMC11273006

[13] Zhao S Dong S Qin Y Wang Y Zhang B Liu A . Inflammation index SIRI is associated with increased all-cause and cardiovascular mortality among patients with hypertension. Front Cardiovasc Med. (2023) 9:1066219. doi: 10.3389/fcvm.2022.1066219 36712259 PMC9874155

[14] Li Z Liu Q Feng Y Li Z Zhang Y Jiang S . Blood glucose mediation of the association between SIRI and mortality in T2DM complicated with ischemic stroke. Sci Rep. (2026) 16(1):5785. doi: 10.1038/s41598-026-36789-4 41559404 PMC12894997

[15] Chen K Li S Xie Z Liu Y Li Y Mai J . Association between oxidative balance score, systemic inflammatory response index, and cardiovascular disease risk: a cross-sectional analysis based on NHANES 2007–2018 data. Front Nutr. (2024) 11:1374992. doi: 10.3389/fnut.2024.1374992 38899319 PMC11186475

[16] Wang R Chen R Tao W Cheng X . Nonlinear associations between the aggregate index of systemic inflammation and cardiovascular disease in adults: evidence from NHANES 2011–2020. BMC Public Health. (2025) 25(1):3031. doi: 10.1186/s12889-025-24320-9 40898106 PMC12406563

[17] Duan C Du Y Chen J Shi S Zhang X Hu Y . Dynamic and static effects of the systemic inflammatory response index on all‐cause mortality in individuals with atherosclerotic cardiovascular disease: evidence from national health and nutrition examination survey. Mediators Inflammation. (2025) 2025(1):5343213. doi: 10.1155/mi/5343213 40270516 PMC12017944

[18] Pengfei C Lui M Zhang L Chen C Wang T Ailin H . Association of SII and SIRI with incidence of cardiovascular disease in cardiovascular-kidney-metabolic syndrome: a prospective cohort study. Front Nutr. (2025) 12:1661826. doi: 10.3389/fnut.2025.1661826 41368184 PMC12683910

[19] Wei X Zhang Z Wei J Luo C . Association of systemic immune inflammation index and system inflammation response index with clinical risk of acute myocardial infarction. Front Cardiovasc Med. (2023) 10:1248655. doi: 10.3389/fcvm.2023.1248655 37711556 PMC10498290

[20] Liu Y Liu J Liu L Cao S Jin T Chen L . Association of systemic inflammatory response index and pan-immune-inflammation-value with long-term adverse cardiovascular events in ST-segment elevation myocardial infarction patients after primary percutaneous coronary intervention. J Inflammation Res. (2023) 16:3437–54. doi: 10.2147/jir.s421491 37600225 PMC10438435

[21] Li Y Zhu N Li X . Systemic inflammatory response index: a novel predictor for cardiovascular disease risk in patients with primary biliary cholangitis. Eur J Med Res. (2025) 30(1):1245. doi: 10.1186/s40001-025-03490-7 41419965 PMC12717740

[22] Chen Y Lian W Wu L Huang A Zhang D Liu B . Joint association of estimated glucose disposal rate and systemic inflammation response index with mortality in cardiovascular-kidney-metabolic syndrome stage 0–3: a nationwide prospective cohort study. Cardiovasc Diabetol. (2025) 24(1):147. doi: 10.1186/s12933-025-02692-x 40158167 PMC11955130

[23] Qin P Ho FK Celis-Morales CA Pell JP . Association between systemic inflammation biomarkers and incident cardiovascular disease in 423,701 individuals: evidence from the UK biobank cohort. Cardiovasc Diabetol. (2025) 24(1):162. doi: 10.1186/s12933-025-02721-9 40234895 PMC12001404

[24] Lin K Lan Y Wang A Yan Y Ge J . The association between a novel inflammatory biomarker, systemic inflammatory response index and the risk of diabetic cardiovascular complications. Nutrition Metab Cardiovasc Dis. (2023) 33:1389–97. doi: 10.1016/j.numecd.2023.03.013 37156671

[25] Zheng H Wu K Zheng H Chen G Lan Y Chen S . High systemic inflammation response index and increased cardiovascular risk and mortality in MASLD: a prospective cohort study. JHEP Rep. (2025) 7(12):101602. doi: 10.1016/j.jhepr.2025.101602 41321936 PMC12657734

[26] Kaur R Kaur M Singh J . Endothelial dysfunction and platelet hyperactivity in type 2 diabetes mellitus: molecular insights and therapeutic strategies. Cardiovasc Diabetol. (2018) 17(1):121. doi: 10.1186/s12933-018-0763-3 30170601 PMC6117983

[27] Esser N Legrand-Poels S Piette J Scheen AJ Paquot N . Inflammation as a link between obesity, metabolic syndrome and type 2 diabetes. Diabetes Res Clin Pract. (2014) 105:141–50. doi: 10.1016/j.diabres.2014.04.006 24798950

[28] Guo Z Song S Cheng H Xie C Zhang M Pei M . Combined SHR and SIRI biomarkers predict increased coronary heart disease risk in type 2 diabetes. Biomol Biomed. (2025) 26:441–51. doi: 10.17305/bb.2025.13032 40929739 PMC12533829

[29] Huang Y Yin X Li Z . Impact of systemic immune inflammation index and systemic inflammation response index on all-cause and cardiovascular mortality in cardiovascular-kidney-metabolic syndrome. Eur J Med Res. (2025) 30(1):645. doi: 10.1186/s40001-025-02929-1 40685352 PMC12278538

[30] Zhao X Lu C Song B Chen D Teng D Shan Z . The prevalence and clustering of metabolic syndrome risk components in Chinese population: a cross-sectional study. Front Endocrinol. (2023) 14. 10.3389/fendo.2023.1290855 PMC10751355 38152127

