# Supplementary material for: Association between systemic inflammation response index and risk of major adverse cardiovascular events in adults with and without metabolic syndrome: a prospective cohort study in Shanghai, Pudong
Source: Front Endocrinol (Lausanne). 2026 Jun 16;17:1846528. doi: 10.3389/fendo.2026.1846528 (PMC13314495; doi:10.3389/fendo.2026.1846528)
Supplement: Supplementary file 1 [file DataSheet1.pdf]

Supplementary Material

1 Supplementary Data

In the total population model, the global chi-square test indicated no significant violation (χ^2 = 19.438, df=16, P=5). Among non-MS participants, the global test was non-significant (χ^2 = 14.58, df=12, P=0.27), but age violated the assumption (P=0.03). Notably, among participants with MS, the proportional hazards assumption was fully satisfied, with a non-significant global test ((χ^2=13.56, df=12, P=0.33) , but age also violated the assumption (P=0.023),therefore, an age-time interaction term was incorporated into the analyses to address this violation. Importantly, the SIRI variable met the proportional hazards assumption consistently across all analytic models (total population: P=0.72; non-MS: P=0.99; MS: P=0.59), supporting the robustness of the estimated HRs as average effect measures over the follow-up period. Detailed results of the proportional hazards assumption tests are provided in the Supplementary Figures below.

1.1 Supplementary Figures

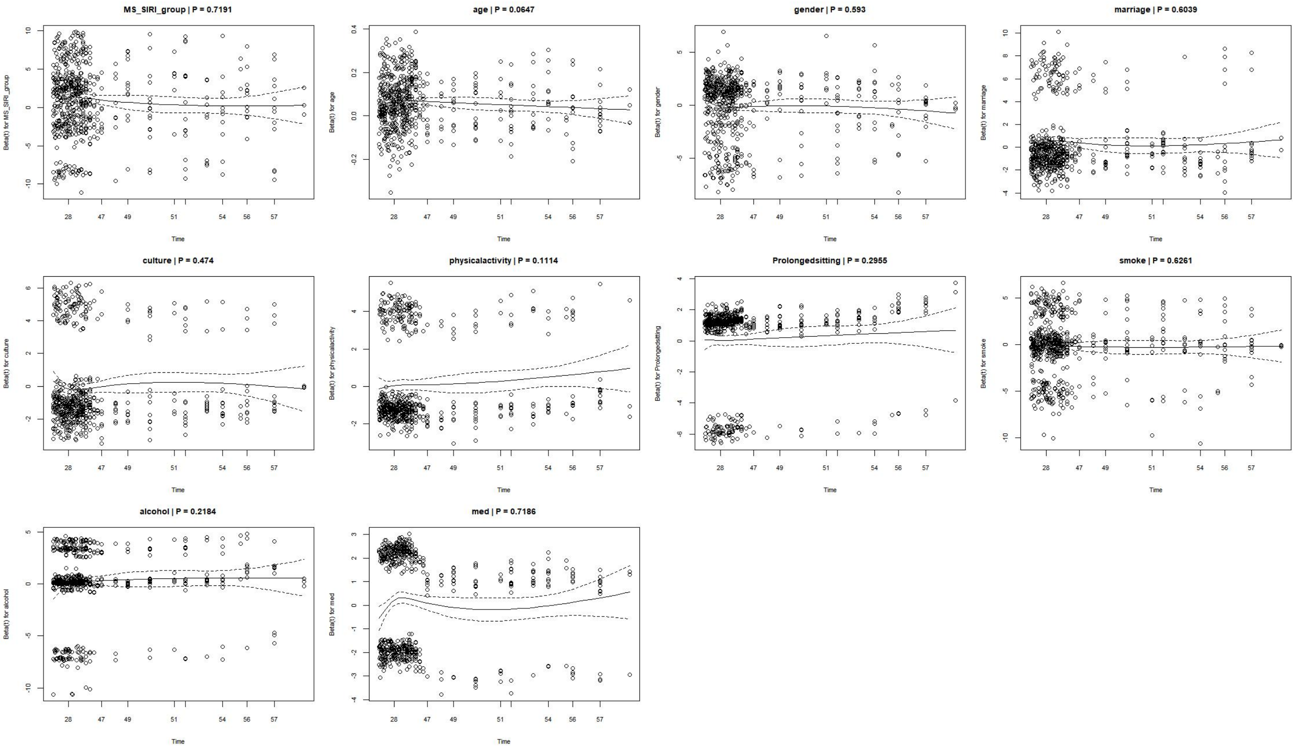

Supplementary Figure 1.Schoenfeld residual plots for the proportional hazards assumption test in the total study population (N=3198)

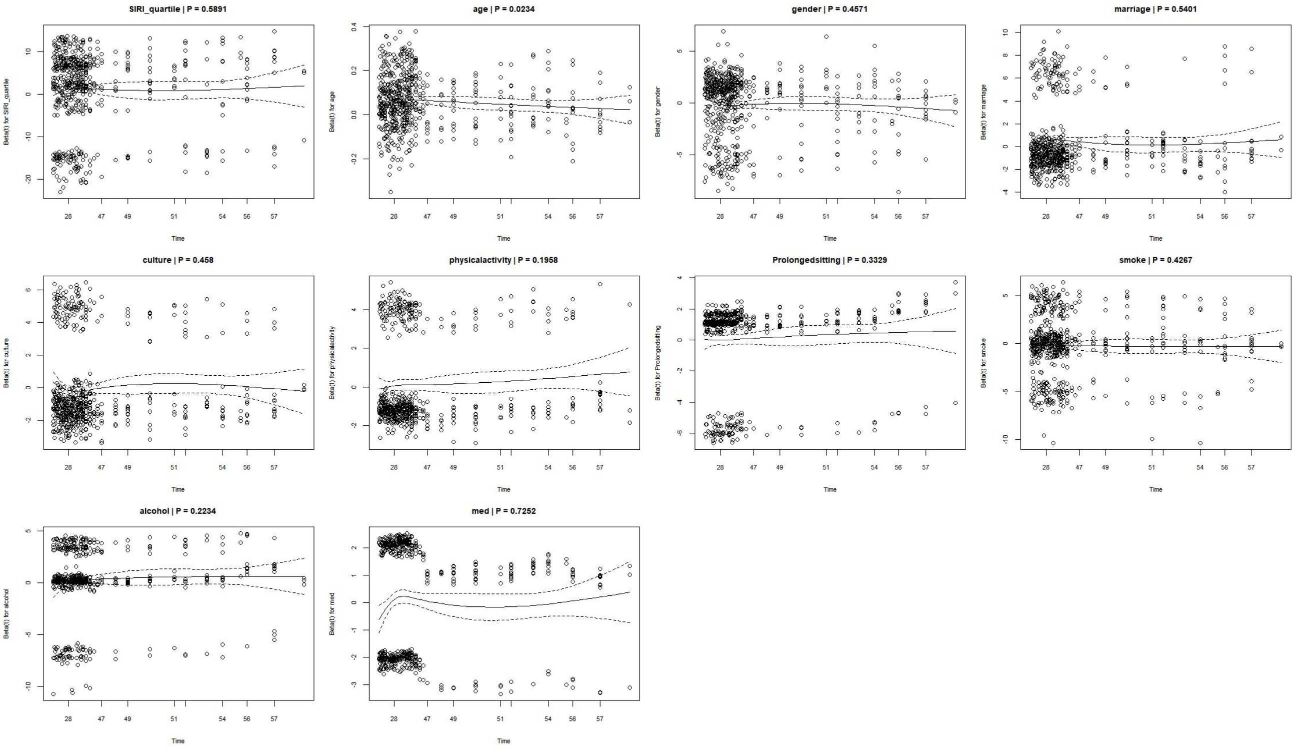

Supplementary Figure 2.Schoenfeld residual plots for the proportional hazards assumption test in the metabolic syndrome (MS) population (n=1318)

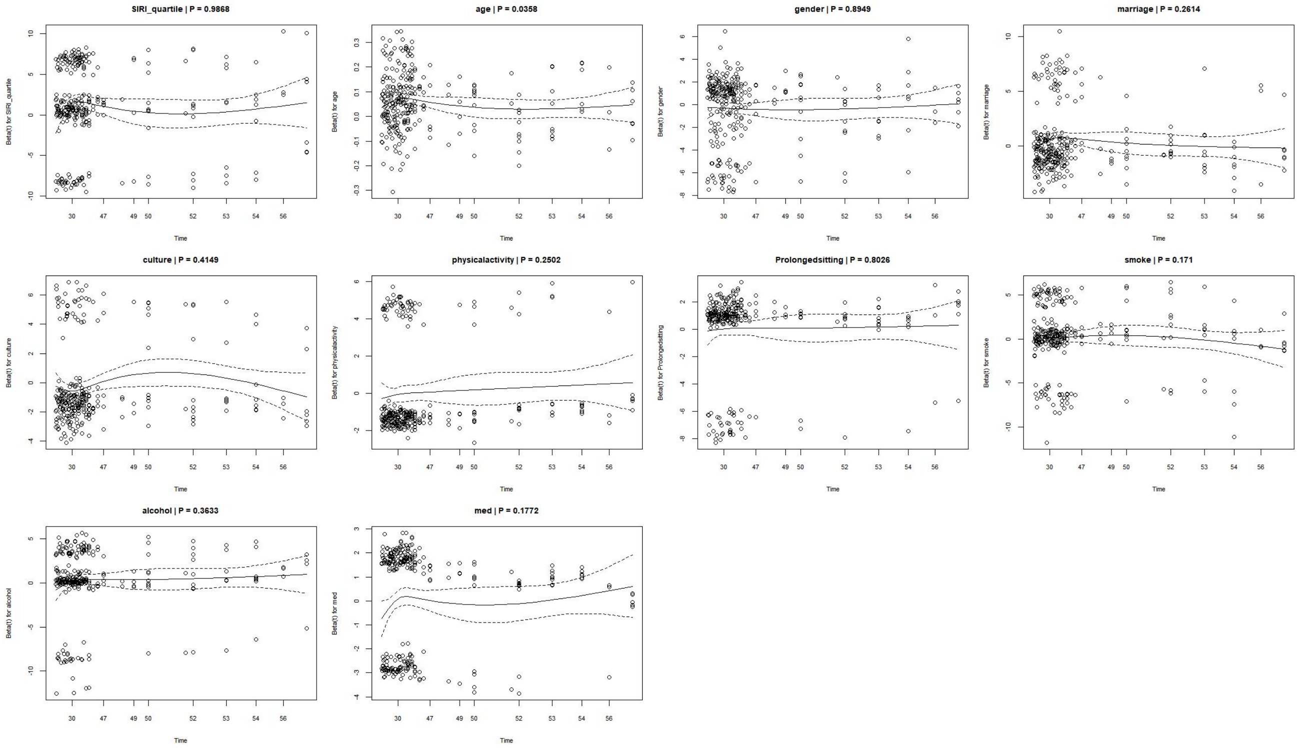

Supplementary Figure 3. Schoenfeld residual plots for the proportional hazards assumption test in the non-metabolic syndrome (non-MS) population (n=1880)

All global and individual Schoenfeld test p-values are presented above each subplot. The solid black line represents the smoothed Schoenfeld residuals, and the dashed lines represent the 95% confidence intervals.
